# Deodorizing Performance of Modified Polyester-Fiber Seamless Knitted Fabrics

**DOI:** 10.3390/ma19163555

**Published:** 2026-08-21

**Authors:** Yani Cai, Jiaying Liu, Miao Su, Zimin Jin

**Affiliations:** 1 College of Textile Science and Engineering (International Institute of Silk), Zhejiang Sci-Tech University, Hangzhou 310018, China; cynwaynel@163.com (Y.C.); liuqyjiayi@163.com (J.L.); kivenjin@163.com (Z.J.); 2 Zhejiang Sci-Tech University Shengzhou Innovation Research Institute, Shengzhou 312400, China

**Keywords:** modified polyester fiber, deodorizing function, seamless knitted fabric, functional textile fibers, structure of the fabric

## Abstract

Bacterial decomposition of sweat produces odors affecting close-fitting garment comfort. This study selected five polyester filament types: coffee carbon polyester filament (CC-PET), bamboo charcoal polyester filament (BC-PET), oyster shell polyester filament (OS-PET), graphene polyester filament (GR-PET), and conventional polyester filament (C-PET). These four yarns exemplify three mainstream deodorizing mechanisms: BC-PET relies on physical adsorption through its porous structure; CC-PET combines adsorption with antibacterial moisture management to suppress odor at the source; GR-PET and OS-PET inhibit bacteria via reactive radicals from oxygen-containing groups and calcium oxide, respectively. Three structures were tested: weft flat knit, 1 × 1 rib, and 1 + 3 false rib. 1 + 1 rib and 1 + 3 false rib differ markedly in elasticity, thickness, and hand. Plain jersey is smooth, soft, and breathable with good extensibility. 1 + 1 rib delivers superior transverse elasticity and dimensional stability. 1 + 3 false rib is loftier and stiffer with enhanced shape retention and thermal insulation. Fifteen specimens were knitted on a seamless circular machine and evaluated using ammonia adsorption rate and acetic acid adsorption rate. Results show that fiber type significantly influences deodorizing performance, with the graphene polyester filament with the 1 + 3 false rib structure achieving the best adsorption for both gases. This provides a theoretical foundation for deodorizing functional fabric development.

## 1. Introduction

**Figure 1:**
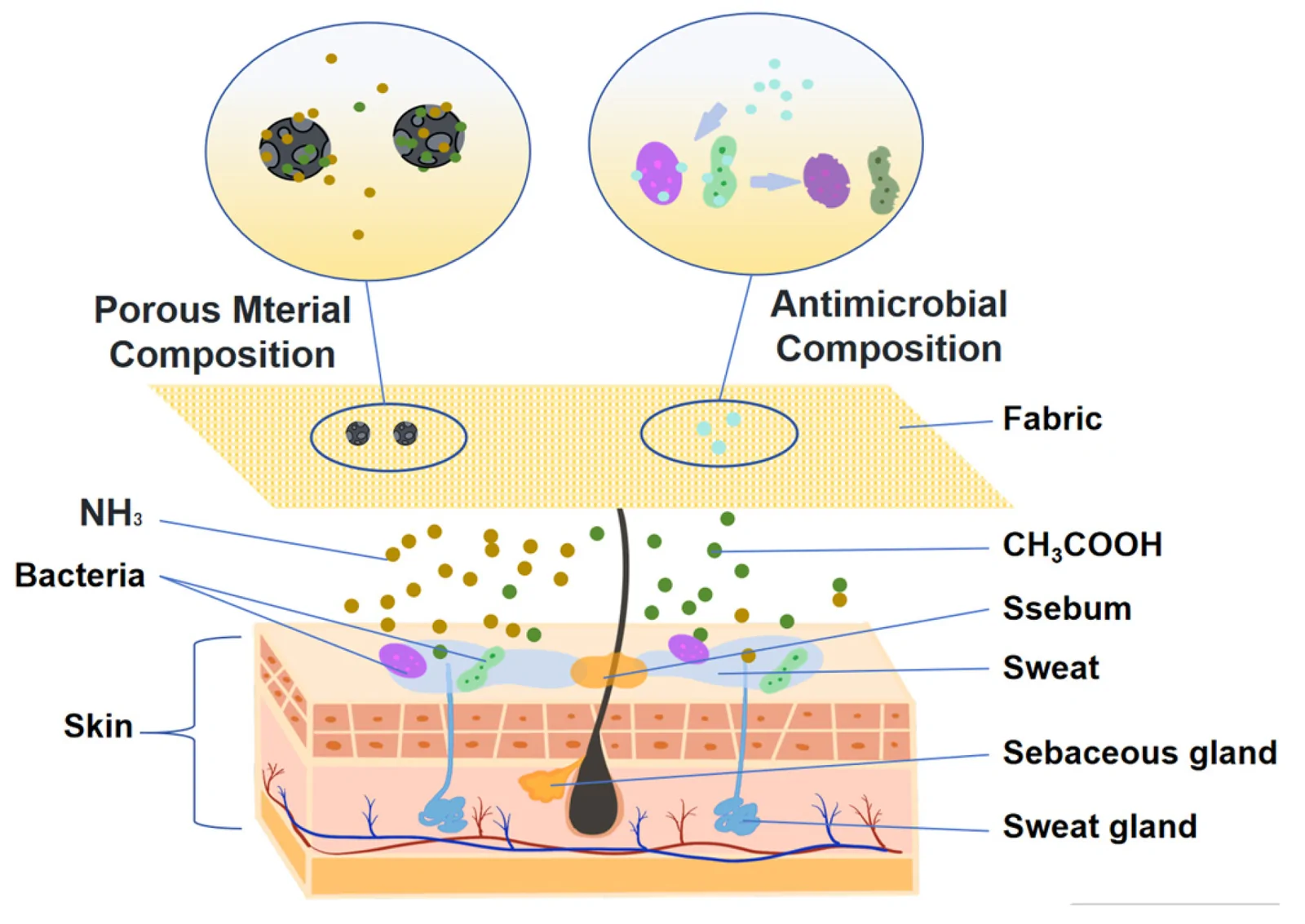
Schematic illustration of the mechanisms of physical adsorption and antimicrobial inhibition.

Body odor generated from human-skin secretions under microbial decomposition is a critical issue affecting wearing comfort and hygiene safety. Volatile organic compounds (VOCs) such as ammonia, acetic acid, and isovaleric acid produced by the bacterial metabolism of sweat and other secretions not only generate unpleasant odors but also promote the excessive proliferation of bacteria [1]. Therefore, developing functional textiles with a deodorizing function has become an important research direction in the field of apparel materials. Currently, textile deodorizing technologies are mainly classified into several categories: physical adsorption, antibacterial source inhibition, chemical decomposition or odor masking, and photocatalytic decomposition [2]. Physical adsorption and antibacterial inhibition are the two most widely applied deodorizing technologies [3]. Their mechanisms of action are illustrated in Figure 1.

Physical adsorption removes odor molecules primarily by increasing the specific surface area of fibers or incorporating porous materials. Bamboo charcoal fiber is a typical representative of physically adsorptive deodorizing fibers. Foo M B et al. [4] investigated the application of bamboo charcoal particles in woven fabrics and found that it has certain effects on fabric comfort. Vuthiganond et al. [5] applied bamboo charcoal to cotton fabrics through printing processes, significantly improving their deodorizing ability. A market report by GII Research [6] also indicated that bamboo charcoal fiber has broad application prospects globally. Spent coffee grounds are a novel and abundant waste resource [7,8], with the new yarn type coffee carbon fiber containing coffee carbon particles in its porous structure, which can not only adsorb odors but also effectively absorb moisture from the body surface, thereby inhibiting bacterial proliferation and reducing odor generation.

Antimicrobial inhibition targets the source by killing bacteria attached to fabrics through functional materials or natural substances, thereby fundamentally preventing the bacterial metabolic reproduction process and inhibiting the generation of odor gases. Both graphene fiber and oyster fiber eliminate odors by incorporating masterbatches with antimicrobial deodorizing functionality. Graphene fibers contain a large number of oxygen-containing functional groups (–COOH, –OH) [9], which produce active free radicals through peroxide reactions; these radicals destroy cellular internal structures and cause cell inactivation, achieving an antimicrobial effect that suppresses bacterial proliferation and thus achieving odor elimination [10,11]. Oyster fibers contain calcium oxide (CaO) and calcium carbonate (CaCO_3_) [12], of which calcium oxide serves as an effective antimicrobial agent and similarly exerts bacteriostatic and deodorizing functions [13].

Fundamental research on deodorizing functional fibers has accumulated substantially [14,15,16,17,18,19,20], yet the existing literature predominantly focuses on single-fiber materials or singular deodorizing mechanisms. This study constructs a three-dimensional evaluation system integrating “fiber type, fabric structure, and odor molecule,” with emphasis on close-fitting garment applications produced via seamless circular knitting processes, aiming to provide theoretical foundations and experimental evidence for the development of seamless knitted underwear from deodorizing functional polyester fibers.

## 2. Materials and Methods

### 2.1. Materials

#### 2.1.1. Selected Yarn Types

This study focuses on investigating the deodorizing performance of seamless knitted fabrics made from various polyester filaments with different deodorizing properties. The face yarns were uniformly selected as four types of functional yarns with a specification of 8.33 tex (75 D): coffee carbon polyester filament (CC-PET), bamboo charcoal polyester filament (BC-PET), oyster shell polyester filament (OS-PET), graphene polyester filament (GR-PET), and conventional polyester filament (C-PET). A control group consisting of a conventional polyester filament with the same specification was also established to ensure data comparability.

The selection of these four types of yarns is based on their representation of three mainstream deodorizing mechanisms: bamboo charcoal fiber belongs to the physical adsorption type, which adsorbs odor molecules through its porous structure [21,22]; coffee carbon fiber combines physical adsorption with hygroscopic antibacterial properties, reducing odor generation at the source; graphene fiber and oyster shell fiber belong to antibacterial source inhibition, which exert antibacterial and deodorizing effects through oxygen-containing functional groups generating reactive radicals and calcium oxide components, respectively. The differences in deodorizing performance among different mechanisms under practical applications will be comprehensively evaluated. The specifications of the face yarns are shown in Table 1.

Seamless knitted fabrics require both lining yarn and face yarn to be woven together during the knitting process. Therefore, the lining yarn selected was a polyamide/spandex covered filament produced by Yiwu Huading Polyamide Co., Ltd, Yiwu, China. with a specification of 2.22 tex (20 D)/2.22 tex (20 D), which utilizes the performance advantages of polyamide and spandex to enhance the elasticity and comfort of seamless knitted fabrics [23].

#### 2.1.2. Fabric Structure Design

The stitch structure of seamless knitted fabrics directly influences their deodorizing functionality and wear performance. Based on research requirements and practical production needs, this study selected three common fabric structures: weft flat knit, 1 + 1 rib, and 1 + 3 false rib.

These three knitting structures represent, respectively, the basic single-face stitch, elastic double-face structure, and modified single-face stitch. They exhibit significant differences in extensibility, elasticity, thickness, and appearance, and are mainstream production structures for seamless underwear and sportswear. The selection of these three categories facilitates systematic investigation of the influence of fabric structure on functional textile performance. Weft plain stitch (plain jersey), as a single-face weft-knit structure, features a simple construction with high production efficiency; the resulting fabric is smooth and soft with good extensibility and breathability, making it suitable as a basic carrier for functional fibers. The 1 + 1 rib stitch is a double-face weft-knit structure characterized by alternating front and back stitch wales; it possesses excellent transverse elasticity and recovery properties, resists curling, and demonstrates superior dimensional stability compared to a plain jersey, making it appropriate for body-contact areas. 1 + 3 false rib belongs to the modified plain stitch single-face structure, producing raised-and-recessed longitudinal stripes; its fabric structure is loftier and stiffer with stronger size retention capability, offering advantages in thermal insulation and visual appearance.

This study adopted a two-factor full-factorial experimental design to systematically investigate the influence of yarn type and fabric structure on fabric properties. Yarn type consisted of 5 levels: oyster fiber polyester filament, graphene fiber polyester filament, coffee carbon fiber polyester filament, bamboo charcoal fiber polyester filament, and regular polyester filament. Fabric structure consisted of 3 levels: plain jersey, 1 + 1 rib stitch, and 1 + 3 false rib. The specific sample plan is shown in Table 2.

#### 2.1.3. Basic Specifications of Seamless Knitted Fabric Specimens

The basic specification parameters of fabric are important indicators for evaluating knitted fabric properties, and their specifications are influenced by yarn type and fabric structure. This study selected density, mass per unit area (g/m^2^), and thickness as the key basic indicators for testing. The results are shown in Table 3.

### 2.2. Methods

This study selected 15 anti-odor polyester-fiber fabric specimens to test their deodorization performance. The 15 specimens underwent ammonia gas adsorption tests and acetic acid adsorption tests to systematically analyze the influence of fabric type and structure on deodorization performance. This provides experimental basis for the development of seamless knitted products with deodorization performance.

Test Instruments: 1000 mL vacuum filtration flask, rubber stopper, hose, manual gas sampler (ZG-1, Beijing Beike Lvzhou Safety Environment Technology Co., Ltd., Beijing, China), adhesive tape, pipette, etc.

Test Materials: 10 cm × 10 cm specimens, 28% ammonia water (analytical reagent, Shanghai Aladdin Technology Co., Ltd., Shanghai, China), 5–100 mg/m^3^ ammonia detector tube (Beijing Beike Lvzhou Safety Environment Technology Co., Ltd., Beijing, China), ≥99.5% glacial acetic acid (analytical reagent, Shanghai Aladdin Technology Co., Ltd., Shanghai, China), 81-type acetic acid detector tube (GASTEC Corporation, Ayase, Japan).

The adsorption rate is calculated as Equation (1).

(1) $$W=\frac{C_{0}-C}{C_{0}}\times 100\%$$

where *W* represents the adsorption rate (%); *C*_0_ represents the concentration of odorous gas 2 h after diluent addition (mg/m^3^); *C* represents the concentration after specimen addition (mg/m^3^).

#### 2.2.1. Ammonia Gas Adsorption Rate Test

A measured amount of ammonia water was injected into a sealed vacuum filtration flask and allowed to volatilize for 2 h. The specimen was then placed into the sealed flask and held until the color development of the detector tube stabilized, at which point the value was read and recorded to monitor the ammonia gas concentration changes. According to GB/T33610.1-2019 [24], if the specimen’s ammonia gas adsorption rate is ≥70%, it is judged to possess deodorization performance.

**Figure 2:**
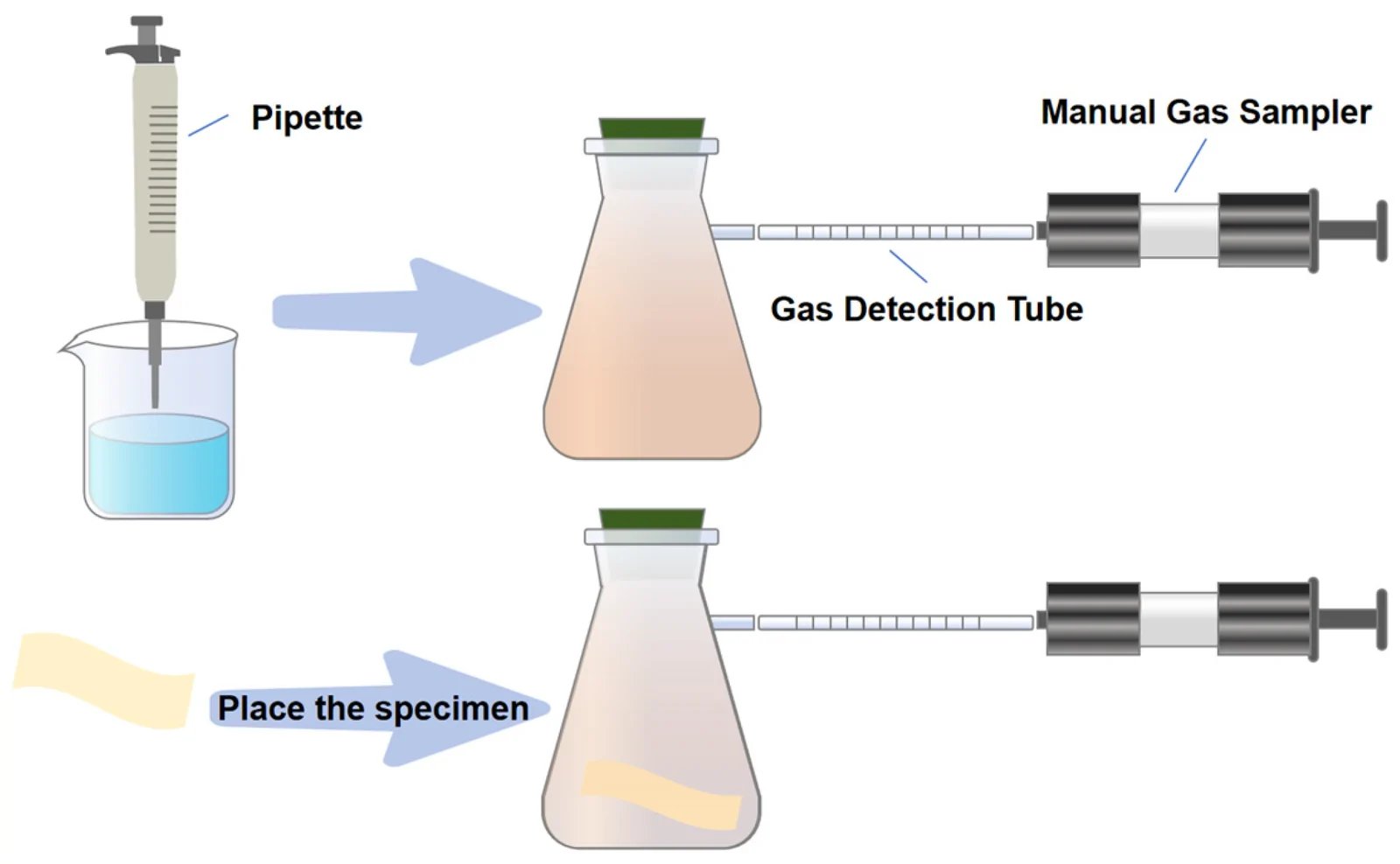
Flowchart of ammonia gas testing experiment.

The experimental procedure is shown in Figure 2.

**Figure 3:**
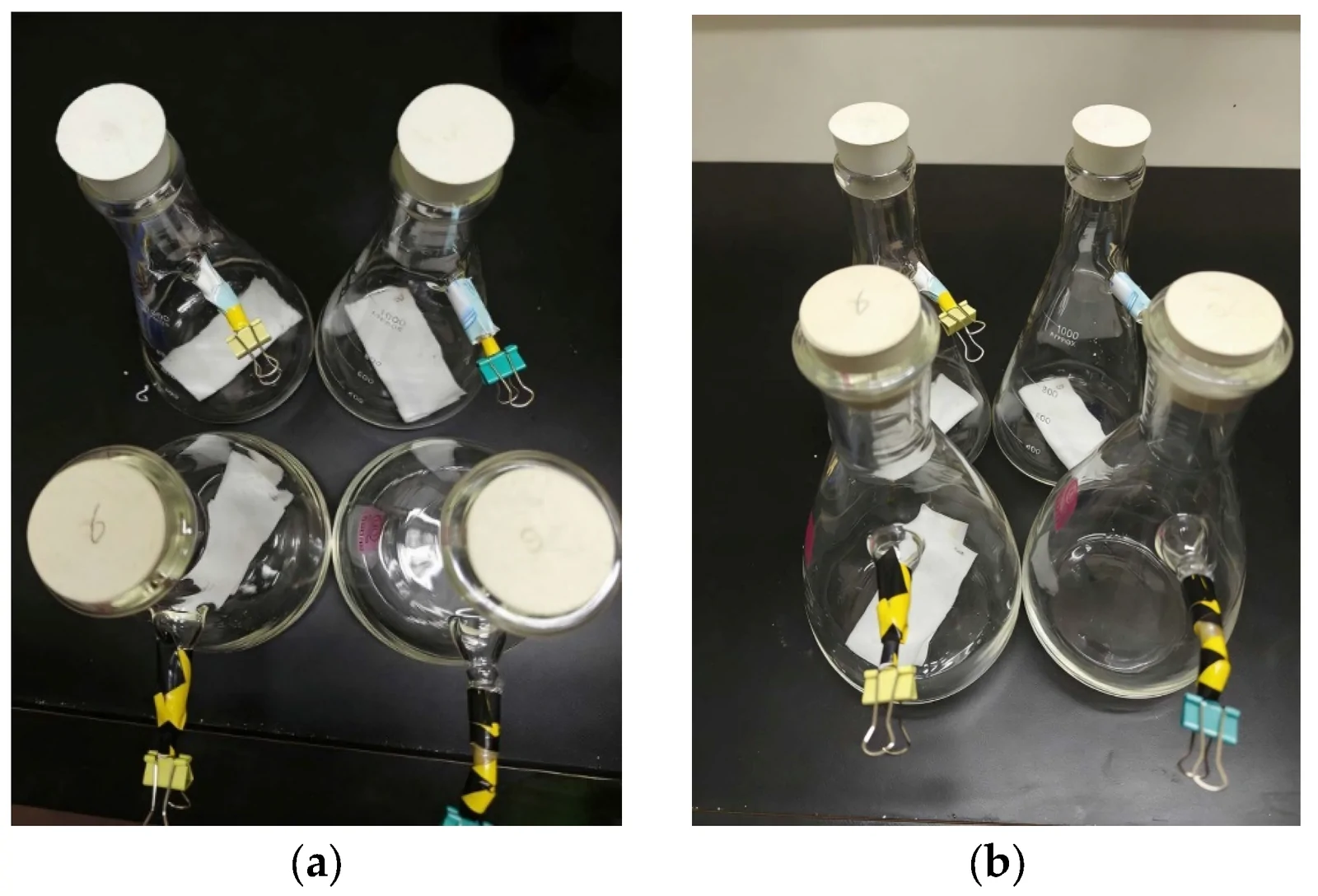
Photograph of fabric odorous gas adsorption rate testing apparatus: (**a**) ammonia adsorption rate test; (**b**) acetic acid adsorption rate test.

Before testing, the flask was cleaned and dried, and all specimens were conditioned under standard atmospheric conditions (RH (65.0 ± 4.0) %, Temp. (20.0 ± 2.0) °C) for 24 h. Figure 3a shows the ammonia gas adsorption apparatus, including a 1000 mL vacuum filtration flask, manual gas sampler, and gas detection tube. The flask was cleaned and dried. A 0.02 mL sample of analytical grade ammonia water was pipetted, diluted to 1 mL with deionized water, and mixed thoroughly. Then, 0.05 mL of the mixture was injected into the flask, sealed with a rubber stopper and connected to a hose. The flask stood for 2 h to allow ammonia volatilization and stable concentration.

Initial ammonia concentration was measured with a gas detection tube. One end of the tube connected to the gas sampler, the other inserted into the hose outlet and taped. Constant low-speed suction of 100 mL gas was performed. After the color of the detector tube had stabilized, the reading was recorded. The conditioned specimen was placed into the flask and resealed. After 120 min, secondary sampling followed the same procedure. Each experiment was repeated three times. Data were calculated using Equation (1) to determine ammonia adsorption rates, thus evaluating fabric adsorption performance.

#### 2.2.2. Acetic Acid Adsorption Rate Test

Acetic acid was injected into a sealed vacuum filtration flask and volatilized for 2 h. The specimen was placed in the flask, sealed, and held for a fixed duration. Acetic acid concentration changes were measured via gas detection tube. According to GB/T33610.1-2019 [17], acetic acid removal rate ≥ 80% indicates deodorization performance.

Experimental method followed ammonia adsorption testing (Section 2.2.1). Figure 3b shows the apparatus: 1000 mL vacuum filtration flask, manual gas sampler, and detector tube. A pipette measured 0.05 mL glacial acetic acid (≥99.5%), diluted 20-fold with deionized water. Then, 0.05 mL was injected into the flask, sealed with a rubber stopper, and connected to a hose with tape. The flask stood for 2 h to attain stable acetic acid gas concentration.

Initial concentration was measured using a gas detection tube connected to the gas sampler, with 100 mL gas suctioned at a constantly low speed. After color stabilized, readings were recorded. The conditioned specimen was placed in the flask and resealed. After 120 min, secondary sampling followed the same procedure. Each experiment was repeated three times. Data were calculated using Equation (1) to determine acetic acid adsorption rates, thus evaluating fabric adsorption performance.

## 3. Results and Discussions

### 3.1. Analysis of Ammonia Gas Adsorption Rate Test Results

**Figure 4:**
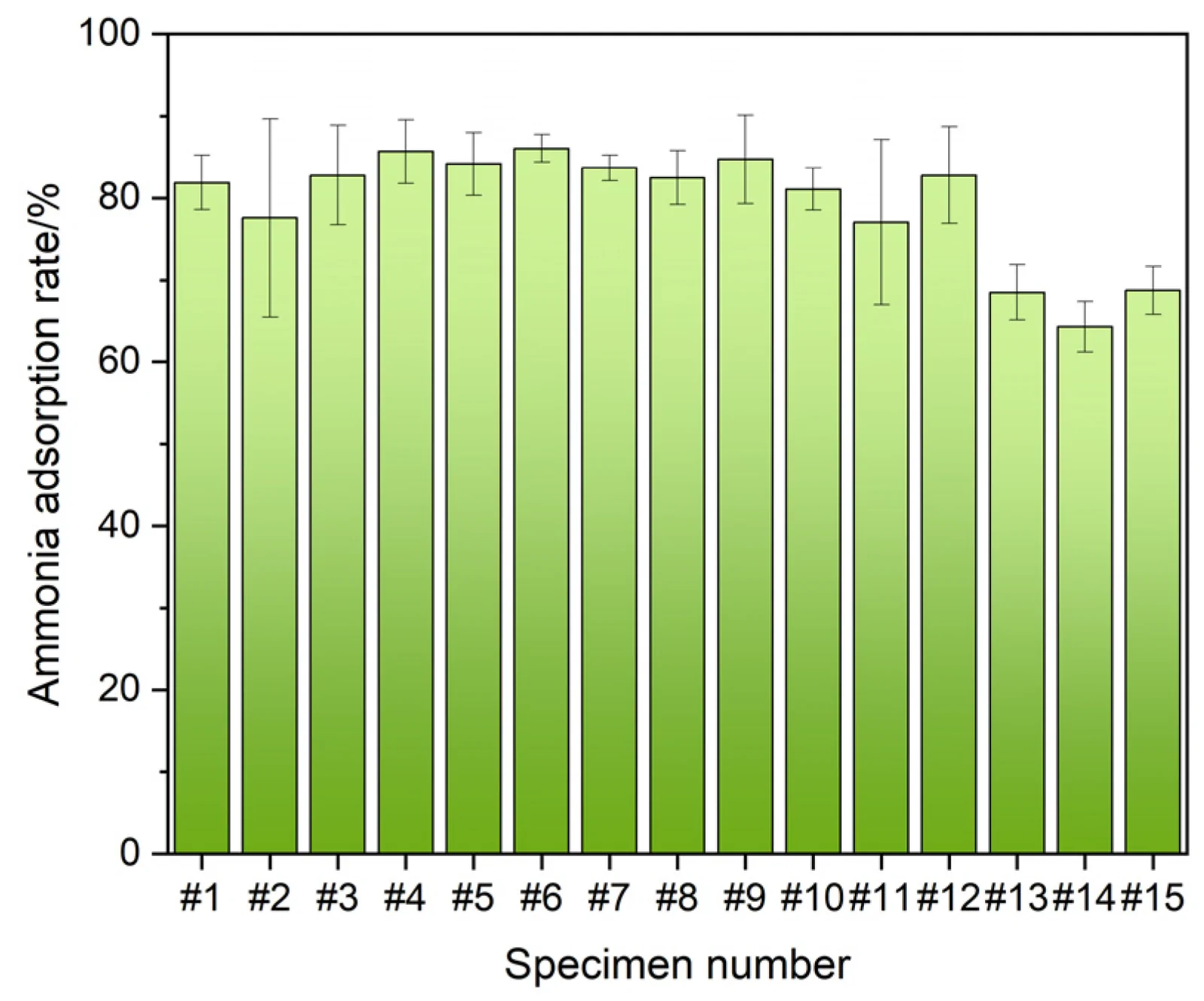
Fabric ammonia adsorption rate test results.

The ammonia adsorption rate results of 15 specimens obtained by the above experimental method are shown in Figure 4.

To further investigate the effects of yarn type and fabric structure on fabric ammonia adsorption rate, a two-way ANOVA was performed using IBM SPSS Statistics (version 32.0; IBM Corp., Armonk, NY, USA). The results are shown in Table 4. From the between-subjects effects test results, the significance *p*-value for yarn type is less than 0.001, indicating that yarn type has a highly significant impact on ammonia adsorption. The significance *p*-value for fabric structure equals 0.001, indicating that fabric structure has a very significant impact on ammonia deodorization performance.

Differences between yarn type and fabric structure were analyzed based on Duncan’s multiple range test, with results shown in Table 5 and Table 6.

Regarding yarn type comparison in Table 5, the five types of yarns were divided into three subsets. Polyester filament (ordinary) was located in subset 1, with a significance *p*-value of 1.000, showing significant difference from the other 4 yarn types, and the lowest adsorption rate. Bamboo charcoal polyester filament and oyster fiber polyester filament were at the same level in subset 2, with no statistically significant difference between them (*p* = 0.606 > 0.05), but both were significantly higher than ordinary polyester filament. Coffee carbon fiber polyester filament and graphene fiber polyester filament were at the same level in subset 3, with no statistically significant difference between them (*p* = 0.073 > 0.05), but both were significantly higher than the first two subsets.

Regarding the fabric structure comparison in Table 6, the three fabric structures were divided into two subsets with significantly different ammonia adsorption rates. 1 + 1 rib structure had the lowest adsorption rate with a mean value of 77.11%, forming subset 1 separately. Plain knitting (80.17%) and 1 + 3 fake rib (81.02%) were at the same level, with no statistically significant difference between them (*p* = 0.214 > 0.05), but both were significantly higher than 1 + 1 rib structure, located in subset 2.

**Figure 5:**
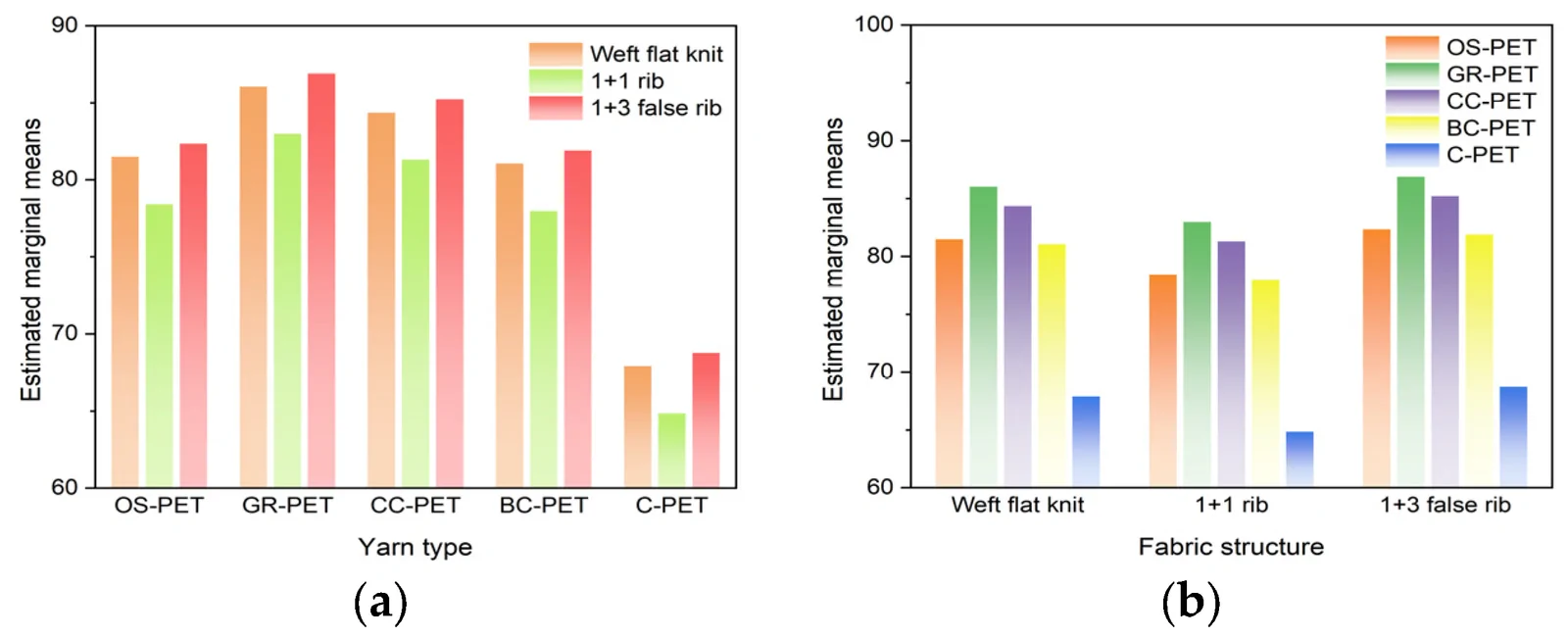
Estimated marginal means of ammonia gas adsorption rate of textile fabrics: (**a**) the independent variable is yarn type; (**b**) the independent variable is fabric structure.

Effect of yarn type and fabric structure on ammonia adsorption rate of fabric, with estimated marginal means shown in Figure 5.

Regarding yarn type, graphene fiber polyester filament exhibited the highest ammonia deodorization level, followed by coffee carbon fiber polyester filament, oyster fiber polyester filament, and bamboo charcoal fiber polyester filament. The last three fiber types showed comparable ammonia deodorization performance, slightly lower than that of graphene fiber polyester filament, while ordinary polyester filament ranked significantly at the bottom. Ordinary polyester filament possesses no acidic functional groups, small specific surface area, and hydrophobic surface, resulting in poor adsorption capacity. Graphene fiber with residual carboxyl groups (–COOH) on the surface can undergo acid–base neutralization with ammonia gas; combined with its large specific surface area (physical adsorption via π-π conjugated structure), it demonstrates optimal adsorption performance. Coffee carbon fiber with honeycomb porous structure and large specific surface area primarily relies on physical adsorption and antibacterial effect, showing secondary performance.

Regarding fabric structure, the 1 + 3 false rib structure achieved the maximum estimated marginal mean value, demonstrating the optimal ammonia elimination effect, followed by the 1 + 1 rib fabric, while the plain knit structure showed the poorest performance. Plain knit structure is lightweight and thin with uniform pores, but the gas retention time is relatively short, resulting in insufficient contact between ammonia molecules and fibers. The 1 + 3 false rib structure features surface irregularities and abundant pores; with loose fabric organization and maximum volume per unit area, it facilitates ammonia molecule adsorption and diffusion. The 1 + 1 rib structure is tight, but the interlocking loops form micropore structures that increase contact area. Therefore, both structures demonstrated superior deodorization performance compared to plain knit organization.

### 3.2. Analysis of Acetic Acid Adsorption Rate Test Results

**Figure 6:**
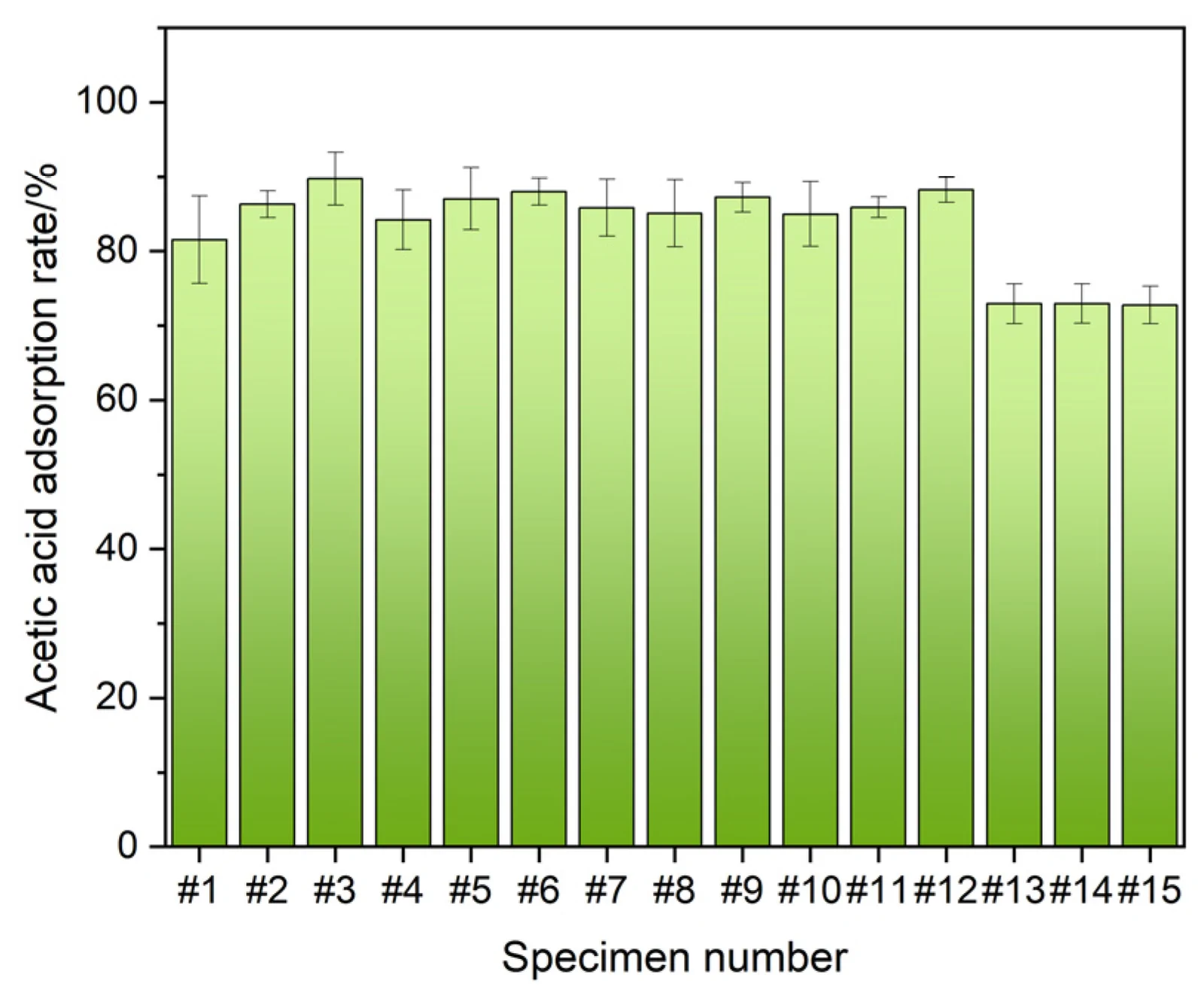
Fabric acetic acid adsorption rate test results.

The results of acetic acid adsorption rate for 15 groups of samples tested by the experimental method described above are shown in Figure 6.

To further investigate the effects of yarn type and fabric structure on the acetic acid adsorption rate of fabric, a two-way ANOVA was performed using SPSS, with the results shown in Table 7.

According to the tests of between-subjects effects table, the significance *p*-value of yarn type is less than 0.001, indicating that the effect of yarn type on acetic acid deodorization is extremely significant. The significance *p*-value of the fabric structure is 0.042, suggesting that the effect of fabric structure on acetic acid deodorization is significant. Among these factors, yarn type plays a dominant role in affecting acetic acid adsorption rate.

Duncan’s multiple range test was conducted to analyze the differences between yarn type and fabric structure, with the results shown in Table 8 and Table 9.

In Table 8, regarding yarn type comparison, the five types of yarn were divided into two subsets based on acetic acid adsorption rate. Ordinary polyester filament was located in Subset 1, with its adsorption rate significantly lower than that of the other four functional fiber polyester filaments. Oyster fiber, coffee carbon fiber, bamboo charcoal fiber, and graphene fiber polyester filaments were located in Subset 2, among which no significant difference existed among the four yarn types (*p* = 0.708 > 0.05), but all were significantly higher than ordinary polyester filament.

In Table 9 regarding fabric structure comparison, the 3 fabric structures were divided into 2 subsets. Plain knit and 1 + 1 rib were located in Subset 1, with no significant difference within the group (*p* = 0.181 > 0.05). The 1 + 1 rib and 1 + 3 false rib were located in Subset 2, with no significant difference within the group (*p* = 0.139 > 0.05). However, a significant difference existed between plain knit and 1 + 3 false rib.

**Figure 7:**
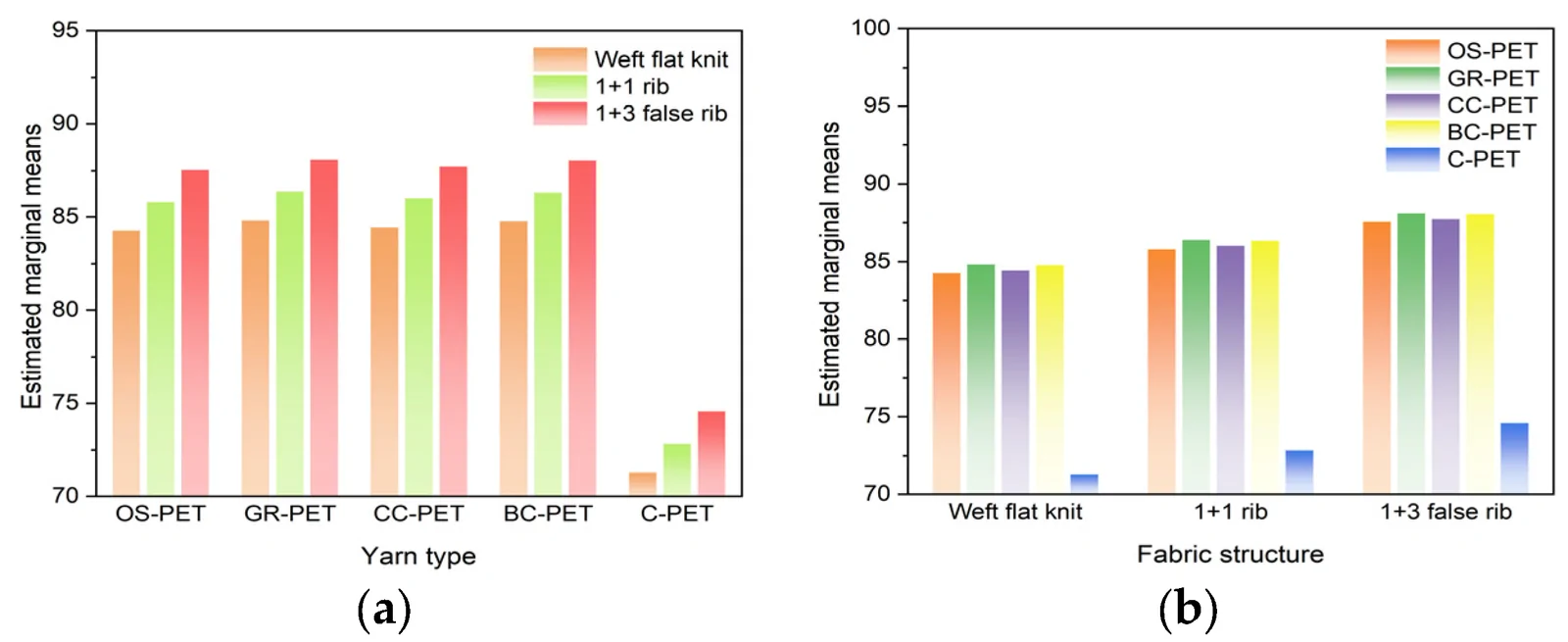
Estimated marginal means of acetic acid adsorption rate of textile fabrics: (**a**) the independent variable is yarn type; (**b**) the independent variable is fabric structure.

Effect of yarn type and fabric structure on acetic acid adsorption rate of fabric, with estimated marginal means shown in Figure 7.

Regarding yarn type, graphene fiber polyester filament and bamboo charcoal polyester filament exhibited higher acetic acid deodorization levels, with comparable performance between the two. Coffee carbon fiber polyester filament and oyster fiber polyester filament followed as the next tier. Ordinary polyester filament ranked significantly at the bottom. Graphene fiber utilizes carboxyl groups and hydroxyl groups on its surface to adsorb acetic acid through acid–base neutralization and hydrogen bonding interactions, demonstrating optimal performance. Bamboo charcoal fiber possesses a large specific surface area with abundant micropores throughout its interior; when contacting acetic acid, it effectively adsorbs the molecules through van der Waals forces into the pores, achieving rapid deodorization effects at levels comparable to graphene fiber. Coffee carbon fiber relies primarily on physical adsorption; after high-temperature carbonization, it lacks alkaline functional groups, resulting in weak chemical neutralization capability. Oyster fiber contains calcium oxide and calcium carbonate that can undergo neutralization reactions with acetic acid, but the reaction is limited to surface-exposed particles while internal particles become ineffective due to polyester encapsulation. The performance of both coffee carbon fiber and oyster fiber is similar. Ordinary polyester lacks active sites and possesses a hydrophobic surface, resulting in significantly inferior performance.

Regarding fabric structure, the estimated marginal mean value for 1 + 3 false rib fabric was the highest, indicating optimal acetic acid elimination effect. 1 + 1 rib fabric demonstrated second-place acetic acid deodorization performance, while plain knit showed the poorest results. The yarns in 1 + 3 false rib fabric undergo significant deformation, forming numerous tortuous microchannels that expose more adsorption sites and achieve the largest specific surface area. Simultaneously, under the same area conditions, 1 + 3 false rib fabric exhibits the maximum volume, enabling more compounds capable of reacting with acetic acid, thereby yielding the highest acetic acid adsorption rate. Although 1 + 1 rib features a tight structure with uniform longitudinal voids, its channels are relatively regular in shape, resulting in secondary adsorption rates. Plain knit exhibits minimal yarn constraint with large straight voids, demonstrating the weakest physical interception capacity and the lowest adsorption rate.

## 4. Conclusions

This study employed the detection tube method to measure adsorption efficiency of ammonia gas and acetic acid, and investigated the effects of yarn type and fabric structure on deodorization performance of 15 polyester seamless knitted fabrics.

In ammonia adsorption efficiency tests, both yarn type and fabric structure significantly affected adsorption efficiency. Regarding yarn type, the ranking was: graphene fiber polyester filament > coffee carbon fiber polyester filament > oyster fiber polyester filament > bamboo charcoal fiber polyester filament > ordinary polyester filament. Graphene fiber polyester filament possesses large specific surface area and can undergo acid–base neutralization with ammonia, enabling efficient ammonia molecule adsorption. Ordinary polyester filament lacks acidic functional groups, resulting in poor adsorption performance. Regarding fabric structure, the ranking was: 1 + 3 false rib > 1 + 1 rib > plain knit. 1 + 3 false rib exhibits surface undulations with abundant pores and a loose structure, achieving maximum volume under the same area, which facilitates ammonia molecule adsorption and diffusion. The plain knit structure is thin and lightweight with relatively short gas retention time, leading to insufficient contact between ammonia molecules and fibers.

In acetic acid adsorption efficiency tests, both yarn type and fabric structure also significantly affected adsorption efficiency. Regarding yarn type, the ranking was: graphene fiber polyester filament > bamboo charcoal fiber polyester filament > coffee carbon fiber polyester filament > oyster fiber polyester filament > ordinary polyester filament. Graphene fiber polyester filament, through acidbase neutralization, hydrogen bonding interactions, and large specific surface area, enhanced both physical adsorption and chemical bonding with acetic acid molecules, substantially increasing the adsorption rate to above 85%. Regarding fabric structure, the ranking was: 1 + 3 false rib > 1 + 1 rib > plain knit. 1 + 3 false rib, with abundant pores and a large specific surface area, facilitates gas penetration and adsorption, resulting in significantly superior acetic acid adsorption performance compared to plain knit.

Yarn type and fabric structure critically affect deodorization performance. Graphene fiber polyester outperforms others in both ammonia and acetic acid adsorption due to large specific surface area, acid–base neutralization, and hydrogen bonding. 1 + 3 false rib is optimal due to abundant pores and large specific surface area. Thus, combining graphene fiber polyester with 1 + 3 false rib offers the best deodorization performance.

## Figures and Tables

**Table 1:** Raw yarn materials and specifications.

Yarn Type	Yarn Specification	Yarn Name	Supplier
Yarn	8.33 tex (75 D)	Coffee Carbon Polyester Filament	Taicang Fangke Textile Co., Ltd. (Taicang, China)
		Bamboo Charcoal Polyester Filament	Taicang Fangke Textile Co., Ltd. (Taicang, China)
		Oyster Shell Polyester Filament	Taicang Fangke Textile Co., Ltd. (Taicang, China)
		Graphene Polyester Filament	Taicang Fangke Textile Co., Ltd. (Taicang, China)
		Conventional Polyester Filament	Yiwu Teda Textile Co., Ltd. (Yiwu, China)

**Table 2:** Fabric specimen scheme.

Specimen No.	Raw Yarn Materials	Fabric Structure
#1	Oyster Shell Polyester Filament	weft flat knit
#2	Oyster Shell Polyester Filament	1 × 1 rib
#3	Oyster Shell Polyester Filament	1 + 3 false rib
#4	Graphene Polyester Filament	weft flat knit
#5	Graphene Polyester Filament	1 × 1 rib
#6	Graphene Polyester Filament	1 + 3 false rib
#7	Coffee Carbon Polyester Filament	weft flat knit
#8	Coffee Carbon Polyester Filament	1 × 1 rib
#9	Coffee Carbon Polyester Filament	1 + 3 false rib
#10	Bamboo Charcoal Polyester Filament	weft flat knit
#11	Bamboo Charcoal Polyester Filament	1 × 1 rib
#12	Bamboo Charcoal Polyester Filament	1 + 3 false rib
#13	Conventional Polyester Filament	weft flat knit
#14	Conventional Polyester Filament	1 × 1 rib
#15	Conventional Polyester Filament	1 + 3 false rib

**Table 3:** Transverse density, longitudinal density, thickness, and mass per unit area of fabric.

Specimen No.	Density/(Courses·(5 cm)^−1^)		Thickness/mm	Mass per Unit Area/(g·m^−2^)
	Courses	Stitches		
#1	96	190	0.77	247.96
#2	115	120	1.122	309.43
#3	147	145	1.932	427.95
#4	96	192	0.754	252.65
#5	115	118	1.122	311.12
#6	144	139	1.932	426.40
#7	97	190	0.752	246.30
#8	117	120	1.134	312.62
#9	147	146	2.02	424.48
#10	96	187	0.76	247.39
#11	119	115	1.12	311.60
#12	148	145	1.958	425.48
#13	98	185	0.81	264.10
#14	116	124	1.144	323.09
#15	141	140	1.824	425.18

**Table 4:** Between-subjects effects test for fabric ammonia adsorption rate.

Source	Type III Sum of Squares	Degrees of Freedom	Mean Square	F	Significance
Corrected Model	656.500 ^a^	6	109.417	110.405	<0.001
Yarn Type	614.241	4	153.560	154.947	<0.001
Fabric Structure	42.258	2	21.129	21.320	0.001
Error	7.928	8	0.991		
Revised total	664.428	14			

^a^. R^2^ = 0.988 (adjusted R^2^ = 0.979).

**Table 5:** Comparison of ammonia adsorption rate among different yarn types.

Yarn Type	N	Subset		
		1	2	3
Conventional Polyester Filament	3	67.1758		
Bamboo Charcoal Polyester Filament	3		80.3141	
Oyster Shell Polyester Filament	3		80.7510	
Coffee Carbon Polyester Filament	3			83.6241
Graphene Polyester Filament	3			85.3007
Sig.		1.000	0.606	0.073

**Table 6:** Comparison of ammonia adsorption rate by fabric structure.

Fabric Structure	N	Subset	
		1	2
1 + 1 rib	5	77.1108	
weft flat knit	5		80.1691
1 + 3 false rib	5		81.0195
Sig.		1.000	0.214

**Table 7:** Tests of between-subjects effects on acetic acid adsorption rate of textile fabrics.

Source	Type III Sum of Squares	Degrees of Freedom	Mean Square	F	Significance
Corrected Model	451.179 ^a^	6	75.197	26.931	<0.001
Yarn Type	424.221	4	106.055	37.982	<0.001
Fabric Structure	26.958	2	13.479	4.827	0.042
Error	22.338	8	2.792		
Revised total	473.517	14			

^a^. R2 = 0.953 (adjusted R^2^ = 0.917).

**Table 8:** Comparison of yarn type on acetic acid adsorption rate of textile fabrics.

Yarn Type	N	Subset	
		1	2
Conventional Polyester Filament	3	72.9000	
Oyster Shell Polyester Filament	3		85.8733
Coffee Carbon Polyester Filament	3		86.0533
Bamboo Charcoal Polyester Filament	3		86.3800
Graphene Polyester Filament	3		86.4333
Sig.		1.000	0.708

**Table 9:** Comparison of fabric structure on acetic acid adsorption rate of textile fabrics.

Yarn Type	N	Subset	
		1	2
weft flat knit	5	81.9180	
1 + 1 rib	5	83.4660	83.4660
1 + 3 false rib	5		85.2000
Sig.		0.181	0.139

## Data Availability

The original contributions presented in this study are included in the article. Further inquiries can be directed to the corresponding author.

## Abbreviations

The following abbreviations are used in this manuscript:

CC-PET	Coffee carbon polyester filament
BC-PET	Bamboo charcoal polyester filament
OS-PET	Oyster shell polyester filament
GR-PET	Graphene polyester filament
C-PET	Conventional polyester filament
